# Colored Petri Net Modelling and Evaluation of Drone Inspection Methods for Distribution Networks

**DOI:** 10.3390/s22093418

**Published:** 2022-04-29

**Authors:** Aleksandra Fedorova, Viktar Beliautsou, Armin Zimmermann

**Affiliations:** 1 Department of Computer Science and Automation, Technische Universität Ilmenau, 98693 Ilmenau, Germany; armin.zimmermann@tu-ilmenau.de; 2 Department of Mechanical Engineering, Technische Universität Ilmenau, 98693 Ilmenau, Germany; viktar.beliautsou@tu-ilmenau.de

**Keywords:** UAV, drone, VTOL, modelling, simulation, colored Petri net, maintenance, life cycle

## Abstract

The UAV industry is developing rapidly and drones are increasingly used for monitoring industrial facilities. When designing such systems, operating companies have to find a system configuration of multiple drones that is near-optimal in terms of cost while achieving the required monitoring quality. Stochastic influences such as failures and maintenance have to be taken into account. Model-based systems engineering supplies tools and methods to solve such problems. This paper presents a method to model and evaluate such UAV systems with coloured Petri nets. It supports a modular view on typical setup elements and different types of UAVs and is based on UAV application standards. The model can be easily adapted to the most popular flight tasks and allows for estimating the monitoring frequency and determining the most appropriate grouping and configuration of UAVs, monitoring schemes, air time and maintenance periods. An important advantage is the ability to consider drone maintenance processes. Thus, the methodology will be useful in the conceptual design phase of UAVs, in monitoring planning, and in the selection of UAVs for specific monitoring tasks.

## 1. Introduction

Unmanned aerial vehicles (UAVs) or drones are aircraft without a pilot on board.

Modern UAVs used for civilian tasks are a young field of technology that is rapidly developing. The IEEE Standard for Drone Applications Framework [1] introduces several typical application fields, including agriculture, electrical engineering, mapping, aerial photography, logistics, and remote sensing. The most common commercial application of drones is aerial photography [2], which is an integral part of facility monitoring. Relatively low cost, increased frequency of inspections, high efficiency and the ability to install various equipment are the main advantages of this method [3,4].

Drones are not just for commercial and military purposes; they are also a hobby and a sport. According to the data published by the Federal Aviation Administration (FAA) of the USA [5], in September 2021 there were already 865,828 drones including 342,357 commercial drones registered. That means 60% of civilian drones are not used for commercial purposes. On the one hand, such accessibility allows the industry to grow rapidly; on the other hand, it leads to a lack of standardised documentation and systematic information. The capabilities of drones and their safe use is an upcoming research area, and new applications of drones are being developed. However, the choice of the right UAV is complicated by little experience with specific models or components. System operators and designers are faced with selecting the best-fitting UAV system design and layout, equipment to be installed, inspection methods, and a host of other factors. Such a choice requires expert knowledge that often only UAV manufacturers have.

Moreover, applications that involve multiple UAVs that operate in a common setup or cooperatively obviously involve additional design choices that are even more complex to decide. This often involves questions of how many UAVs are necessary as minimum equipment to fulfil the required tasks, how many ground stations are necessary and where to place them, which UAV should be assigned to what task, etc. Apart from such strategic system design questions, other decisions have to be made online during run time, similar to scheduling or flight path issues and decisions about whether a UAV should continue its task or return to a base. These are classic optimization questions in systems design under uncertainty, which should be supported by models, evaluation methods, and software tools.

In this paper, we propose a method for modelling and evaluating multidrone inspection systems for distribution networks. Such networks can include oil and gas pipelines, electricity distribution networks, or even roads. We consider as UAVs fixed-wing aircraft (planes), vertical take-off and landing (VTOL) aircraft, such as multirotor systems (quadcopters or multicopters) and convertiplanes, which are combinations of planes and multicopters. Models for such systems should be easy to understand and thus visual while also being capable enough to cover the necessary object types and attributes as well as their behaviour. For these reasons, we chose stochastic coloured Petri nets [6] as the basic description. Petri nets allow for modelling systems containing parallel running processes naturally. They are represented as bipartite graphs with directed arcs and visualize systems and processes. The behaviour of a system using drones is influenced by many stochastic factors, such as weather, the probability of breakdown, the need of maintenance and others. Stochastic Petri nets [7] allow us to take such factors into account and simulate the nature of the model as closely as necessary to reality.

Abstracting away from task-specific algorithms, almost any drone mission comes down to the most efficient flight path. For the type of networks to be monitored that we consider here, routes have to follow the network connections and do not have to cover areas, i.e., linear objects. Consequently, mission performance is most influenced by speed and range.

The actual scientific task, the solution of which is presented in this paper, is to develop and implement a unified model using Petri nets to evaluate the feasibility of a particular drone model for monitoring linear objects. The described modelling method will allow for achieving the required level of abstraction. A modular system will be created that can be adapted to many typical tasks.

The scientific novelty of this research is the proposal of a mathematical model of a continuous UAV monitoring process using coloured Petri nets as well as in developing a preventive maintenance model in accordance with typical UAV specifics.

The structure of the paper is organised as follows. The second section describes an overview of existing solutions and related works. The methods and the design of the model are introduced subsequently. Section 4 evaluates the model and presents numerical results. The paper ends with a conclusion.

## 2. Background

### 2.1. Related Works

In the conceptual design phase, decision-makers are faced with the need to find a compromise solution among hundreds of options [8]. At this stage, it is critical to determine the required characteristics of the aircraft, as changes in the later stages of development will increase rapidly. Several methods exist in systems engineering, e.g., the article [9] describes the Interactive Reconfigurable Matrix of Alternatives (IRMA) method based on morphological analysis. The application of this decision-making concept for long-range aircraft has also been demonstrated. Other methods exist: the Genetic Algorithm [10], Sensitivity Profiling [11], Probabilistic Design Methods [12], variations of Multiple-Criteria Decision Making (MCDM) methods and others. The number of combinations of technical parameters is considerably reduced by using the mentioned methods. Further choices are made based on the goals of aircraft development using simulation methods.

The task of modelling the monitoring of a linear object by drones is very similar to classical tasks in transport logistics. Many mathematical models and software tools were created to implement logistics solutions for transport companies (Oracle transportation management, 1C TRM logistics, Log4Pro, AMB cloud, etc.). However, all of them do not take into account the features of UAVs and cannot be used to model unmanned aviation without significant modifications [13]. For this reason, a distinct type of route-planning task has been assigned specifically to drones.

The task of route planning has been considered by commercial companies as part of the development of UAV software. Typically, these products use coordinates to specify the trajectory that one or a group of drones will follow or specify the area or size of an object to be inspected. Almost every major company has its own solution. For example, one of the industry leaders DJI, offers DJI GS PRO software [14] for route planning. Other companies that focus on UAV software also offer solutions. SPH Engineering has developed the UgCS tool [15] for UAV flight mission planning and control. Similar solutions are developed by the British company Dronecloud [16] and the American company Measure [17]. The subsidiary of the laboratory of computer vision of the Federal Polytechnic School of Lausanne Pix4D [18] has developed a set of software products for unmanned aerial vehicles, including route planning.

The scientific community is constantly proposing new solutions for modelling drone flight missions. The authors of [19] suggest software for a ground control centre. They use Google Maps and geometrics to build a route and take into account the range of the UAV. The authors of the paper [20] use a greedy algorithm based on tagging. A platform for the automated monitoring of environmental parameters using UAVs in agriculture has been tested over the long term and has shown an increase in efficiency compared to other methods [21]. There are also solutions for controlling a group (swarm) of quadcopters [22], including for monitoring large areas [23] or for delivery tasks [24]. Some solutions allow different types of drones to be used in the planning of a single mission [25]. There are also special missions that require more detailed planning and control due to the complexity of the task, for example, the need to change the trajectory of the quadcopter during flight [26] or the peculiarities of military missions [27]. Several review articles [28,29] are published in which the authors make in-depth analyses of existing route-planning algorithms and compare the algorithms and protocols based on performance criteria.

All of the solutions described above are of high practical relevance, and some of them are widespread in the professional environment and offer broad functionality. However, most of them support only certain models or aerodynamic schemes of drones, not allowing for changes in the design, while other studies are purely theoretical, and almost none of the existing solutions allow the use of simulation results for decision making at the conceptual design stage of the monitoring system. In our experience, they consider drone flights as one-time events and attempt to maximise the effects of the missions performed. This approach does not allow the system to be evaluated in a comprehensive way nor the entire UAV life cycle to be taken into account.

### 2.2. Coloured Petri Nets

We chose coloured Petri nets for our approach, which are an extension of classical Petri nets. Petri nets were introduced by Carl Adam Petri as part of his doctoral thesis “Interaction with Automata” [30]. The model in Petri nets is an oriented bidirectional graph that contains two types of vertices: places and transitions, which are connected by directed arcs. Places represent state variables, and transitions represent events. The arcs that lead from a place to a transition are called input arcs. Arcs that lead from a transition to a place are called output arcs. In the graphical representation, places are represented by circles and transitions by rectangles (often of different types to visualize varying timing properties). Places can contain tokens modelling objects. Token movement (i.e., state changes) is associated with firing a transition. A transition is fired when its conditions are met. Multiple tokens can be moved in one firing. Places may have a limit (capacity) to the number of tokens they can hold. Petri nets allow for modelling systems containing parallel processes and are widely used for solving application-specific systems design problems.

There is no distinction between tokens in the basic concept of Petri nets. The use of classical Petri nets for models, in which tokens carry some essential attributes, leads to the cluttering of models, as additional places and transitions have to be deployed to track such attributes (i.e., unfolding). In coloured Petri nets [6,31,32], the tokens have values attached to them, which allow for distinguishing the tokens. Individual token types can be defined, similar to a class definition in an object-oriented programming language. Coloured Petri nets can be used to extend the capabilities of timed and hierarchical nets.

Tokens can belong to different classes, which are distinguished by attributes. Attributes can contain numbers, logical values, text, dates and time. Places can only contain tokens of one class. Transition firing can depend on token attribute values and change them during the firing process. The transition can have different activation and firing modes depending on the input tokens and can contain conditions (guards) that limit the type of tokens that can pass through the transition.

## 3. Materials and Methods

In this section, we describe the assumptions and constraints identified during the analysis of the subject area that formed the basis of our method. We also describe the steps of model development and implementation, as well as performance measures.

### 3.1. Design of the Model

This section introduces a modular Petri net model design for drone inspection systems and provides explanations and examples of its main steps. The model was designed following IEEE Standard for Drone Applications Framework [1]. A general description of the system is given first, followed by a more detailed discussion of its components. In the subsection describing the flight, the characteristics of the UAV and the facility that directly affect the monitoring process are discussed. Then the results of the drone maintenance study are given and the necessary actions to prepare for the flight are discussed. The standard maintenance procedures are described. Finally, the modelling section presents the assumptions and constraints used in the model, the discretisation steps and the method of performance evaluation.

#### 3.1.1. Description of the System

The distribution network to be monitored is assumed to have a graph-like structure with connections and some interconnecting nodes, following our previous experience in assessing pipeline systems. The model represents a monitoring process of such an object by drones. Each UAV performs continuous monitoring of a part of the overall network graph. The speed, range, identification number and number of UAVs are assumed to be known for the modelling step. The monitoring object has at least two checkpoints, one at the beginning, one at the end. An object can have several intermediate checkpoints as well as intersection points. The distance between the closest checkpoints is known. At any checkpoint, the UAV always executes preflight preparations and charges the batteries before take-off.

**Figure 1:**
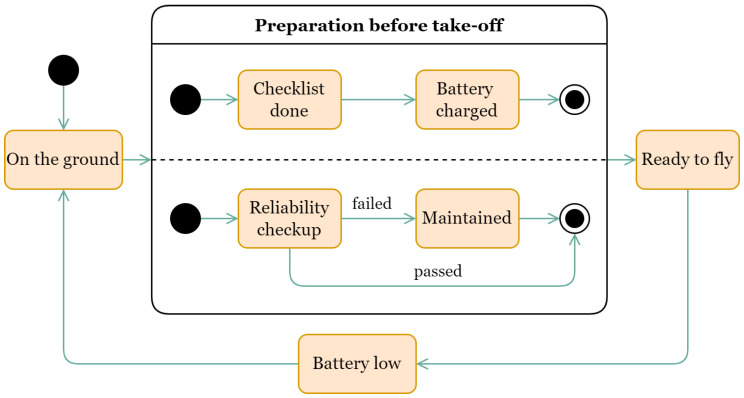
A statechart diagram of the monitoring process.

We assume that the monitoring process is performed in the following order: The UAV takes off at one point, flies while monitoring and lands at another point when the battery is low. The UAV may not land if it has enough range to reach the next checkpoint. When flying forward, the UAV monitors an object and its speed may be limited by the payload capability. When flying to the start of the object, the speed of the UAV is limited only by the specifications of the UAV. The UAV is maintained between flights according to the manufacturer’s recommendations. Figure 1 illustrates the monitoring process described above.

The goal of the later performance evaluation of the model is to determine the highest possible frequency to monitor the whole object when the frequency is understood as the number of flights in a given period (e.g., per day, week or month) similar to typical requirements. Each performance evaluation of a certain model setup thus tells the designer if the given configuration is balanced and fulfils the requirements (see later on).

#### 3.1.2. Drone and Object Characteristics

The drone’s flight along the route is the main element of monitoring. During the mission, the drone observes the object using the payload (i.e., camera and possibly other sensors). The data obtained are stored or processed for further use. The implementation of this part depends on a wide range of parameters. These parameters can be divided into two groups: drone and object characteristics.

According to the IEEE standard [1], the UAV flight performance is characterised by the following drone characteristics:

1. Height: Generally drone flights take place at low altitudes and mostly in automatic mode, as it is limited by the capabilities of the target load and free airspace. This parameter should be considered at the route planning stage.
2. Speed: The monitoring of extended objects can be considered as flying at a constant average speed, depending on the payload or the capabilities of the drone. However, the drone may fly at different speeds at various stages of the mission, for example, while collecting data compared to the flight back to the base station. Thus, it is necessary to take into account average and maximum speed independently.
3. Radius of flight: If several drones of the same type are used in a system, the drones can use each other’s infrastructure, i.e., a return to the take-off point may not be necessary. Therefore, instead of the flight radius, we will use a range that is equal to the doubled flight radius.
4. Endurance: For endurance requirements, we will consider preflight preparations and maintenance.
5. Manoeuvrability of aircraft: The manoeuvrability of the aircraft is taken into account when preparing the flight route.
6. Take-off: Take-off is performed in accordance with UAV specifications.

Hence, the model will consider range, average and maximum speed as general drone parameters.

An individual object to be monitored is of linear structure, such as pipelines, electric power transmission lines or similar, where it is possible to organize checkpoints for drones. The following parameters are important for this model:

1. Checkpoints: places where drones can undergo preflight procedures. A preflight procedure includes checklist execution, battery charging and maintenance if required.
2. Distance: the distance between two nearest checkpoints. Determined not only by the distance on the plane but also by the topography of the surface.
3. Intersection points: points of intersection of several sections of the monitored object.

#### 3.1.3. Preflight Procedures

The general operation process includes checklist execution and battery charging as preflight procedures. The checklist is a procedure regulated by the UAV manufacturer to be carried out before each flight. It is necessary to make sure that all systems are working properly and that no unexpected events will occur during the flight. Depending on the communication system used, drone configuration, payload and other components, the procedure may vary significantly.

However, to simulate this process, we only need to know the time it will take to follow such a checklist for a particular drone.

In addition to the checklist, charging the drone’s batteries is a mandatory procedure to prepare the drone for flight. Almost all drones used for monitoring are equipped with an electric power train. This is primarily due to the convenience and safety of using such a propulsion system. The drone with such a propulsion system uses battery charging for refuelling.

Drones use high-current lithium batteries. The battery is a highly stressed element and needs frequent inspection and testing. For this reason, the batteries in drones are not built-in and must be removed each time they are charged. Three mainly used types of batteries include:

- Li-ion—lithium-ion batteries used for small machines, high capacity with minimum weight.
- Li-Po—lithium polymer batteries used for drones with a high payload. They have the highest current output.
- Li-PoFe4—lithium iron phosphate batteries used for extreme weather conditions such as subzero temperatures.

High C-rate batteries are usually used. The C-rate indicates the measure of a battery’s charge and discharges in relation to its capacity. In other words, 1C-rate means that a battery can be discharged in 1 h, 0.5C-rate in two hours, and 6C-rate in 10 min [33]. It is the same with battery charging. However, a higher C-rate has a negative impact on battery life. Therefore, we will assume that the batteries are charged at 1C-rate, i.e., they will be fully charged in one hour.

A charging time of one hour can be considered reasonable because even if the drone is charged faster, it has to include mandatory procedures such as removing and installing the battery.

Most manufacturers attach graphical illustrations to help with such work on the drone. These materials are called flow charts and are used to help with work or personnel training. If the person operating the drone follows all the guidelines, we can predict in advance how long a particular operation will take. According to the manufacturer’s instructions, all the actions described in the model under development can be specified as the time intervals required to perform each operation.

### 3.2. Maintenance

Maintainability is an important flight platform parameter that influences reliability significantly. In our approach, we will consider preventive maintenance to avoid (most) failures. Since the industry is young, available data on the reliability of drones are insufficient for statistical studies. Thus, it was decided to be guided by the recommendations of drone manufacturers [34], personal experience and communications with industry stakeholders at relevant events. The drone is a modular system. The three most vulnerable modules are propellers, batteries and motors. Manufacturers give the following typical recommendations for replacing these components:

1. Propeller every 20 flights.
2. Batteries every 200 flights.
3. Motors every 400 flights.

#### 3.2.1. Propeller

Propellers are one of those elements that are easy to replace during maintenance. They are usually designed originally as quick-release elements. However, the number of propellers can vary from one to eight or more. In addition, more complicated systems, e.g., for folding propellers, can also be used. We will therefore assume an average propeller replacement time of 30 min.

#### 3.2.2. Batteries

The batteries in the drone should always be easy to replace. This is recommended following operating safety regulations, as batteries are a highly stressed element. The battery replacement process is similar to the battery charging process. The only difference is that instead of charging, the battery should be checked to ensure that it is fully operable and compatible with the drone. In general, this process should not take more than 30 min.

#### 3.2.3. Motors

The motors are an element that must be well-fastened. They can turn the propellers with high frequency. Replacing a motor always requires removing the propeller first, so the time for replacing the motor cannot be shorter than the time for replacing the propeller. The motor mount assembly may contain more than ten components and each element must be inspected for damage. Therefore we will assume that the motors will be replaced within two hours.

#### 3.2.4. Maintenance Frequency

A maintenance period should be determined before the simulation. To summarise the above-mentioned, parts exchange will take 30 min for propellers and batteries and 2 h for motors. Thus, the maintenance period will be calculated by the following formula:

(1) $$t_{\mathrm{mnt}}=n_{\mathrm{flight}}*\left(t_{\mathrm{flight}}+t_{\mathrm{charge}}+t_{\mathrm{prep}}\right)$$

where $n_{\mathrm{flight}}$—the maintenance period of a particular component; $t_{\mathrm{flight}}$—average flight time; $t_{\mathrm{charge}}$—charging time and $t_{\mathrm{prep}}$—preflight checklist execution.

### 3.3. Performance Measures

The performance measure we add to the model should return how often the object can be monitored. For example, a monitoring period of one year is set. The result would represent how many times the object will be inspected during a year. A higher number is better, as it means the UAV will be more often at each point and thus failures are less of the time undetected.

### 3.4. Implementation

In this subsection, the implementation of the model using Petri nets for monitoring and the maintenance model are presented. This section also describes some of the specifics of interacting with the selected modelling tool.

#### 3.4.1. Software

The TimeNET tool was used to implement the model, as it has the necessary feature set. TimeNET [35,36] is a software package for modelling and evaluating Petri nets, where transition times can be exponentially distributed, deterministic or with another distribution function. Models can be defined using a graphical user interface. Result indicators can be defined at the model level using a special syntax.

The advantages are the ability to simulate different types of Petri nets, including coloured Petri nets, syntax checking, code generation, performance analysis capability, a step-by-step simulation function, the so-called “token game” and the ability to automatically generate model components has been added.

Regarding coloured Petri nets, the model class in TimeNET combines generalized stochastic Petri nets (GSPNs) with arc-variable coloured Petri nets. The syntax of inscriptions follows object-oriented programming ideas. The additional information can be found in the book [6] that covers the simulation algorithms, including the pseudocode, and in the paper [37] which briefly describes the tool and has a CPN model example with a more detailed description.

#### 3.4.2. Architecture

**Figure 2:**
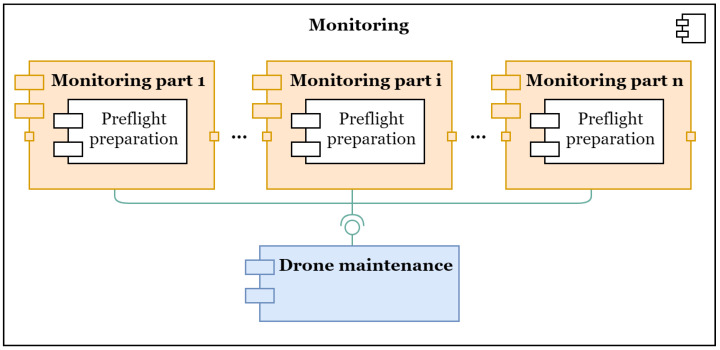
The component diagram of the monitoring model. The component diagram of the monitoring model shows that the drone’s area of responsibility at a particular monitoring object is divided into sections, depending on the possible take-off and drone landing points. Each section is modelled as a separate monitoring module, and they are all linked together in a chain. Furthermore, the monitoring modules are linked to the drone maintenance module, where the components of each drone are modelled in parallel.

Consider the component diagram of the model. Figure 2 shows a component diagram of the modular structure of the model. There are three types of modules in the model: a module that simulates maintenance, a module to simulates the monitoring of one of the segments of the object, and a preflight module that is used in the monitoring module.

The monitoring modules can be connected in series with each other, and all the monitoring modules are connected in parallel with the maintenance module, which provides them with information about the current status of the UAV.

Thus, the model can be adapted to any network structure due to its modularity.

### 3.5. Basic Modules

**Figure 3:**
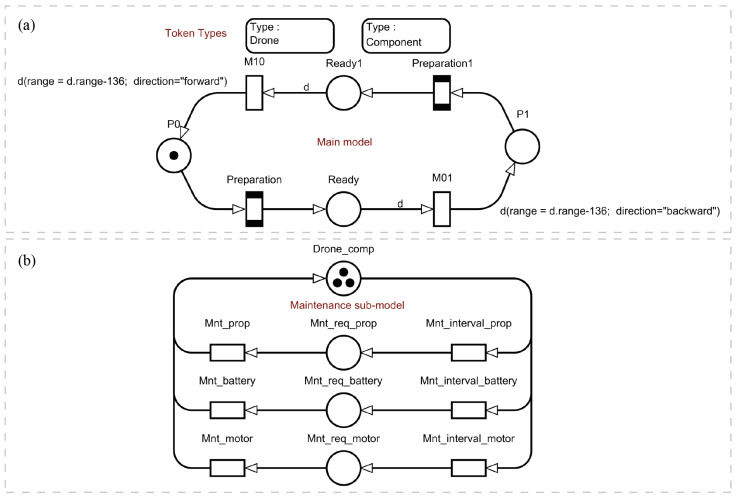
The upper hierarchical level of the model is presented. There are two parts: (**a**) The main monitoring model that simulates the flight of a drone from one point to another, as well as preflight preparation. (**b**) The maintenance model that simulates the life cycle of drone components such as propellers, batteries and motors.

The goal of the work is to create a modular performance evaluation model. The modules should be easily adaptable to real-world tasks. Figure 3 shows the upper levels of the model connected by global conditions. Part (a) of the model simulates the flight of a UAV from one checkpoint to another, while part (b) simulates maintenance.

Two token types are defined in the model. The “Drone” type simulates a UAV. The “Components” type represents the components of the UAV that were chosen for maintenance. The “*Drone*” type has three attributes:

1. “id”—the identification number of the UAV. The data type is an integer. This parameter is needed to independently track the need for maintenance and determine the path for each drone.
2. “range”—the available range. The data type is an integer. This parameter will be used to track the remaining range to decide if a landing is necessary.
3. “direction”—the direction of flight. The data type is a string. The parameter is used to determine the path and the current speed of the drone. In the model, the values “forward” for the direction of flight during monitoring and ”backward” for the direction of flight to return to the beginning of the route can be used.

The type “*Components*” has two attributes:

1. “idD”—the identification number of the UAV. The data type is an integer. The parameter indicates which drone this component corresponds to.
2. “type”—component type. The data type is a string. In the model, the values “props” for propellers, “battery” for batteries and “motor” for motors can be used, as well as any other type of component if its maintenance period is known.

#### 3.5.1. Monitoring Model

The maintenance model is represented in Figure 3b. Places “P0” … “Pn” are checkpoints. Place “Ready” contains the status of the UAV when preflight procedures are completed. Places correspond to the “On the ground” and “Ready to fly” states in the statechart in Figure 1. Both can only contain “Drone” tokens. Transitions “M01” … “Mmn” simulate the flight of the UAV, where m is the number of the take-off point and n is the number of the landing point. A deterministic time function is used. In transitions where tokens move from right to left, it is assumed that the UAV returns to the beginning of the route. In this case, the UAV has a higher speed than during monitoring. This part of the model interacts only with “Drone” type tokens.

It should be explained that the letters above the arrows in Figure 3a represent the variable name assigned to the respective token, which is required to change the token colours. As mentioned above, “Drone” tokens have three attributes (colours): identification number, range and direction, which they carry with them. The colours change after a transition is fired. The colour change rule is described as the parameter of the arrow exiting the transition. In the case shown, we reduce the remaining range by subtracting the length of the section and the direction of travel by assigning a different value.

#### 3.5.2. Submodel Preparation

**Figure 4:**
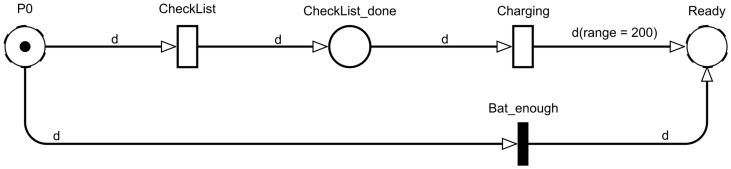
The preflight procedures execution model “Preparation”.

The monitoring model also contains substitutional transitions “Preparation” with a subnet describing the UAV’s preflight procedures. The subnet is shown in Figure 4. Conceptually, the “Preparation” and “Preparation1” submodels differ only in local guards of the transitions, which will be discussed below, so only “Preparation” will be considered.

The “P0” place is the input to the submodel and the “Ready” place is the output. The “CheckList_done” place reflects the state when the UAV has successfully passed the preflight checklist and can only contain tokens of the “*Drone*” type.

The “CheckList” transition simulates the execution of a checklist before take-off. An exponential time function is used. In fact, if this transition fires, it means that the UAV has landed at location “P0”. The “Charging” transition simulates the battery charging process. The exponential time function is used. Transition “Bat_enough” simulates skipping a checkpoint, i.e., the UAV has enough remaining range to reach the next checkpoint and does not land.

#### 3.5.3. Maintenance

The maintenance model is represented in Figure 3b. All places and transitions of this model can only interact with tokens of type “Component”. The “Drone_comp” place models the state when a component of the UAV does not require maintenance. A UAV is considered capable of performing a flight mission when all three components of each particular UAV are simultaneously at the “Drone_comp” place. The “Mnt_req_<type>” locations simulate states when the respective components require maintenance. Transitions “Mnt_interval_<type>” simulate periods between maintenance for each component type. Transitions “Mnt_<type>” simulate the process of maintenance of the respective types of UAV components. An exponential time function is used.

### 3.6. Parameters of the Model

Assume that the distance between points P0 and P1 is 136 km and the speed of the UAV when flying from P0 to P1 is 90 km/h and when flying from P1 to P0 is 140 km/h. Below are tables summarising the model parameters.

#### 3.6.1. Token Types

Table 1 describes the token types. There are two types of tokens used: the first type represents UAVs and the second one represents components of the drones that should be tracked for maintenance. The purpose of each parameter was described in Section 3.4.

#### 3.6.2. Places

In the coloured Petri net, places may contain only one type of token. Table 2 describes token types that places may contain.

#### 3.6.3. Transitions

Transitions differ in type, time function and guards. Most transitions in the model are the usual timed ones, but immediate (firing after zero time) and substitution transitions (which only act as placeholders for low-level refinements) are also used. The deterministic time function was defined for transitions that simulate flight because it was assumed the UAV follows a predefined algorithm that will correct the flight parameters if necessary. Other transitions have an exponential time function. There are two types of guards, global and local. In this model, global guards are used to connect the maintenance model with the monitoring model. Local guards are used for specifying the path of tokens.

### 3.7. Measures

The UAV monitors only when flying from point “P0” to “P1”, which follows from the limitations, as the flight speed significantly affects the quality of the data obtained. Consequently, an equal monitoring interval can be obtained for each object part.

The frequency can be measured directly in the model during the simulation. The result is shown after the simulation. In the TimeNET, it is sufficient to add a measurement of the “M01” transition throughput to do this. Multiplying the result by the required time period in hours, the number of flights in the specified period will be obtained.

The monitoring modules can be connected in series with each other, and all the monitoring modules are connected in parallel with the maintenance module, which provides them with information about the current status of the UAV. The model can be scaled to the route length and the required number of UAVs.

## 4. Results

This section describes a real-world example of the model application. Section 4.1 describes the application problem, Section 4.2 covers the model, and Section 4.3 contains results of the performance evaluation.

### 4.1. Description of the Task

**Figure 5:**
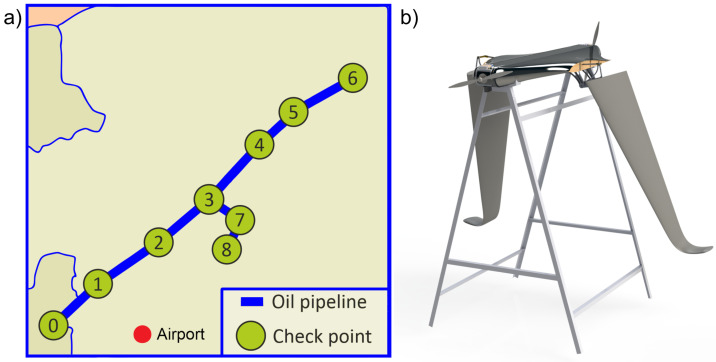
(**a**) The monitoring object—an oil pipeline with 9 checkpoints. (**b**) An example of the UAV of convertible type.

Let us consider a segment of an industrial oil pipeline. A scheme is shown in Figure 5. There are nine pumping stations in total, which will be considered as checkpoints, and the distance between them is given in Table 3. The UAV of type VTOL (vertical take-off and landing) will be used for monitoring, the range is 200 km, the maximum flight speed is 100 km/h, and the maximum allowed flight speed for monitoring is limited by the characteristics of the target load and equals 60 km/h. Three UAVs will be used for monitoring.

### 4.2. Description of the Model

**Figure 6:**
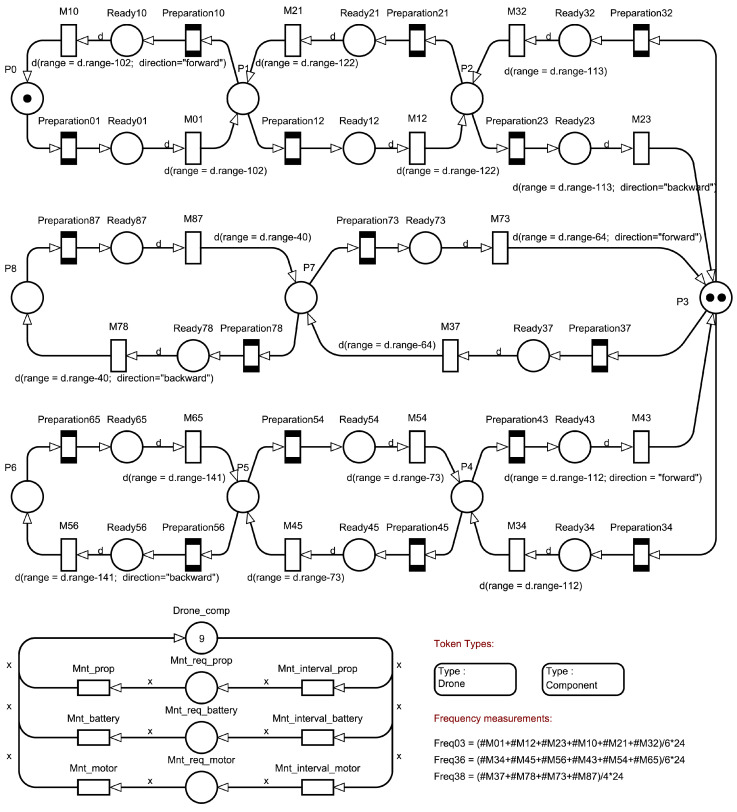
A scheme of the model implemented in TimeNET based on the oil pipeline example.

The main elements of the model are described in Section 3.4. Figure 6 shows a scheme of the model. Eight monitoring segments have been created. The object is divided into three sections with assigned UAVs: P0–P3, P3–P6 and P3–P8. Each UAV is responsible for its own section. At place P0, there is a token with identification number 0 for the UAV which investigates section P0–P3; place P3 contains two tokens, one with identification number 1 for section P3–P6 and the second with identification number 2 for section P3–P8.

The directional conditions of the tokens on the network were restricted as follows:

- Token with ID number 0 only visits places P0,P1,P2 and P3.
- Token with ID number 1 only visits places P3,P4,P5 and P6.
- Token with ID number 2 only visits places P3,P7 and P8.

All transitions simulating the flight have been configured according to the expected flight time for the particular network section.

The “Drone_comp” place contains nine tokens: three for each UAV. Based on the flight time at each section, an average flight time of 1.28 h was determined. The checklist time is 6 minutes. Thus, the periods between maintenance are 47.6 h, 476 h and 952 h for propellers, batteries and motors, respectively.

Monitoring frequency measurements were added for each of the three sections. This frequency can be easily expressed by the mean of the throughput of the transitions (i.e., their firing frequencies) multiplied by the required time period in hours.

### 4.3. Simulation Results

The experiment was conducted on a computer with an Intel Core i5-1035G1 processor at 1.00 GHz and 8.00 GB RAM in a 64-bit Windows 10 environment. A stationary simulation was carried out in TimeNET version 4.5. All other parameters had standard values. Further simulations were carried out under these conditions. Code generation and model compilation took 1 min 54 s including less than one second of simulation time to achieve an accuracy of 5% relative error for a confidence interval of 95%.

The results of the simulation are shown in Table 4. This table shows that the P0–P3 section has the highest number of full monitoring cycles of one flight per day, the P3–P6 section has one flight and the P3–P8 section has 4 flights. A significantly increased number of flights per day at P3–P8 is due to the short distance between checkpoints. Therefore, the UAV saves time on battery charging and checklist execution.

### 4.4. Determining a UAV Grouping Method

As an example of model-based system design improvement, it is possible to check the results obtained for the task described in Section 4.1 by changing the monitoring scheme. In Table 4, the section of the object P3–P8 has the highest monitoring frequency which is almost three times higher. This is an obvious hint of an unbalanced system configuration. It can be explained by the short distances between checkpoints P3–P7 and P7–P8 (Table 3). Assume we do not want to change the number and the type of drones. We will expand section P3–P8 by adding segment P3–P4 or P2–P3 to balance the allocation of resources.

For the first option, the responsibilities of the drones are shared as P0–P3, P4–P6 and P4–P8. The result of the simulation is shown in Table 5. The distribution of the monitoring frequencies has become more balanced, but the minimum frequency is still around 1.5.

For the second option, the responsibilities of the drones are shared as P0–P2, P3–P6 and P2–P8. The result of the simulation is shown in Table 6. With this monitoring scheme, it is possible to increase the minimum frequency to 1.8. This is a typical example of how model-based systems design can be used for a what-if analysis.

Other monitoring scheme options will not increase the frequency of monitoring due to the arrangement of short and long sections. Thus, the second option (P0–P2, P3–P6 and P2–P8) can be considered the most suitable when three drones are used.

The number of UAVs directly affects the frequency of monitoring, and its optimal choice is another typical example of systems design. Let us assume that each section is monitored by one drone. Then the number of drones needed to monitor the whole object will be equal to the number of sections into which it will be divided. In our example application, the object is divided into eight sections.

**Figure 7:**
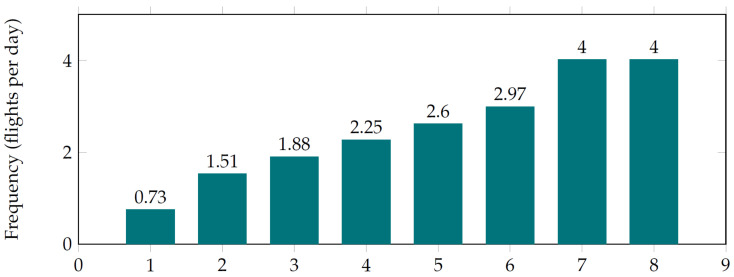
Dependence of monitoring frequency (number of flights per day) on the number of drones.

We can easily vary parameters as well as the number of drones in our model. The results are presented in Table 7 and in Figure 7. Figure 7 represents the dependence of monitoring frequency (number of flights per day) on the number of drones for the whole object. The table represents the number of flights per day on sections between take-off and landing points. Each number corresponds to monitoring by one drone. As a route, the shortest path is assumed, i.e., if the section includes an intersection point, the drone does not visit an additional branch. This is why the lowest frequency of monitoring by one drone in Figure 7 differs from the value in Table 7 corresponding to take-off at point 0 and landing at point 8.

According to the simulation results (Figure 7), if one UAV monitors the entire object, the monitoring frequency will be 0.73 times per day. This frequency is the minimum for the characteristics of the drone specified before. The highest monitoring frequency will correspond to the lowest monitoring frequency of a section, assuming that the object is divided into eight sections. Simulation results for each of these sections are shown in Table 7 in the highlighted cells. The object is branching (Figure 5), so checkpoint 6 is not directly connected to checkpoint 7. Checkpoint 3 is an intersection point; thus, the cell with a value of 9.60 is coloured. Thus, the highest monitoring frequency of the whole object per day is 4.00 when using eight drones. It should also be noted that seven drones will be sufficient to achieve the highest monitoring frequency. This is because the P5–P6 section is the longest, and it is longer than the sum of the lengths of the two shortest adjacent sections P3–P7 and P7–P8.

It can be concluded that the lowest monitoring frequency would be 0.73 flights per day and the highest monitoring frequency of four flights per day could be achieved with seven drones per day. Thus, the model makes it possible to determine the optimum number of drones for a given task. The cost of operations and investments could also be taken into account for more detailed analyses.

### 4.5. Determining a Suitable UAV Configuration

Let us now consider the effect of the range of each UAV on the frequency of object monitoring. For the study, we will choose the monitoring scheme P0–P2, P2–P8 and P3–P6, as it allowed us to achieve the highest monitoring frequency in Section 4.4 when using three drones.

The shortest permissible range of the drone is determined by the longest segment length, taking into account the 5% navigation error, and equals 150 km.

The longest range is limited by the maximum take-off weight of the aircraft including batteries and payload, the transmitter range and the design scheme of the UAV.

Medium and large UAVs with a weight over 25 kg have a very long range. Thus, the range of military UAVs can reach 1200 km and more. However, the use of such UAVs is impossible without a specially equipped runway. Therefore, we will consider small UAVs here.

The record range of a small UAV was established by the company IN-FLIGHT Data Inc. in 2018 in Canada [38]. The range of the UAV of the flying-wing type was 414 km. Thus, we will limit the theoretical permissible range of the UAV to 400 km.

**Figure 8:**
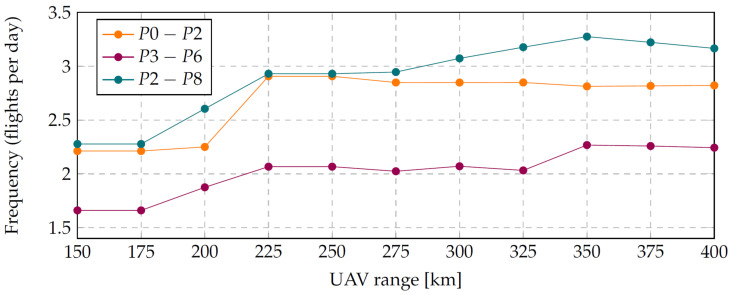
Dependence of the monitoring frequency on the UAV range.

The simulation result for a UAV with a range of 150 to 400 km in increments of 25 km is shown in Figure 8. Increasing the range of the UAV to 225 km allows for increasing the frequency of monitoring the entire object from 1.8 to 2.0 times per day. The next threshold is a UAV range of 350 km. Then, the whole object monitoring frequency would be 2.3. However, such a significant increase in range would be costly in practice.

It can be concluded that the range of a UAV has a significant impact on the monitoring frequency. Since the range of a UAV is highly dependent on other parameters that can be easily changed, such as battery capacity, payload weight, fairings, trajectory and speed, it is not always necessary to change the UAV model to increase the range. Sometimes it is possible to increase the frequency of flight by making the UAV lighter or by installing a larger battery or winglets.

### 4.6. Determining an Expected Amount of Maintenance Cycles

Similar to the definition of flight frequency, the component replacement and maintenance intervals can be determined with the model. By measuring the transition capacity “Mnt_<type>” and multiplying by the time period, the expected number of maintenance cycles can be found. This will determine the expected cost of consumables in drone operation.

Consider the example described above. We use the option of grouping three drones, previously defined as optimal for this object, and set the time interval to one year. Table 8 shows the results of the simulation. It can be seen that by grouping the UAVs in a more balanced way, their components will wear out evenly, which will be a benefit for the equipment’s operation.

## 5. Discussion

After the analysis of the state of the art, we can conclude that most of the existing solutions can be classified into one of three groups:

- Conceptual design, which allows for selecting drone parameters for the task;
- Solving logistical problems, which allows for determining the cost of using certain equipment;
- Route-planning task, which finds the optimal route taking into account the UAV’s characteristics.

Our solution, on the other hand, addresses these tasks in an integrated way and allows us to solve them within a single model. For example, for the conceptual design of a drone, it is possible not only to take into account the technical characteristics required by the object but also to simulate the entire product life cycle at the early design stages.

To solve logistical problems, we can offer an evaluation of different drone grouping and configuration options in terms of drone costs, maintenance as well as human resources. Therefore, our model allows for a more accurate and complete analysis of these parameters, based not only on the technical characteristics of drones but also on external factors.

For the route-planning task, the model allows us to determine the most appropriate location of charging stations or take-off points, vary the number of drones and their parameters and optimise the route in case of intersection points. In addition to the basic route-planning tasks, we can also take into account the endurance of the UAV for the desired trajectory. This will be an advantage in the long-term perspective.

In terms of practical applications, our methodology allows us to replicate mathematically the monitoring process, taking into account the UAV’s characteristics and stochastic factors in UAV operation. We strongly believe that such a model can be used for a wide range of applications. For example, monitoring customers can select the monitoring strategy, type and UAV model for a particular object among the existing commercial solutions to meet their requirements. The result of the methodology will be a clear term of reference, based on the UAV’s capabilities and which suits the customer’s purpose.

The organisation providing the monitoring can evaluate whether their resources are being used properly, adjust flight paths, select equipment, formulate a maintenance strategy, and find ways to improve their performance.

Component and UAV manufacturers can quickly adjust their solutions and developments within the existing elemental base to meet specific practical challenges. Moreover, our model allows for the comparison of conceptually different types of drones, such as planes, multicopters and convertiplanes. The model can be scaled not only to the object of the drone application but also to the aircraft components. Thus, with this method, it is possible to consider the life cycles of all components and optimise the operating cost over the long term.

At the concept stage, it can be assumed that such a method will allow the formulation and introduction of standards in the UAV field, which will increase drone engagement, the safeness of its application as well as the unification of technical solutions.

From a technical point of view, we consider the following ways of further developing this methodology:

- To expand the number of factors taken into account in the monitoring modelling, e.g., to consider weather conditions;
- To automate the process of adding modules and to create a database of UAVs to simplify the research process;
- The model can be developed into a software product, which will be useful for consumers of facility monitoring services, as well as for UAV manufacturers and service providers to assess their competitiveness.

Overall, in order to improve our model, we are interested in real statistical data on the use of UAVs in application tasks. This will allow us to increase the accuracy of the modelling to identify the most important factors and parameters.

## 6. Conclusions

Summarising the work conducted, we propose a method for the modelling and performance evaluation of drone monitoring processes. The proposed method is implemented as a coloured Petri net model describing the flight of each drone in a mission group.

The model combines the processes of route planning, drone preparation for flight, the operational mission model, the UAV wear model, and the maintenance model. Consequently, the model combines existing solutions in this domain. The model created is easily scalable and adaptable to real-world tasks and flexibly covers the features of different UAV types as well as stochastic factors. This has been demonstrated with the modelling of an oil pipeline monitoring process.

## Figures and Tables

**Table 1:** Token types.

Token Type	Attributes	Data Types	Notes
“Drone”	“id”	integer	UAV identification number
	“range”	integer	Remaining range
	“direction”	string	Flight direction
“Component”	“idD”	integer	UAV identification number
	“type”	string	Component type

**Table 2:** Places and token types.

Name	Token Type
P0, P1	Drone
Ready, Ready1	Drone
CheckList_done	Drone
Drone_comp	Component
Mnt_req_prop	Component
Mnt_req_battery	Component
Mnt_req_motors	Component

**Table 3:** The distance between checkpoints.

Take-Off Point	Landing Point	Distance between Points (km)
P0	P1	102
P1	P2	122
P2	P3	113
P3	P4	112
P4	P5	73
P5	P6	141
P3	P7	64
P7	P8	40

**Table 4:** Monitoring frequency in a day.

Section	Monitoring Frequency in a Day
P0–P3	1.484486
P3–P6	1.888126
P3–P8	5.388123

**Table 5:** Monitoring frequency in a day for the configurations P0–P3, P4–P6 and P4–P8.

Section	Monitoring Frequency in a Day
P0–P3	1.504622
P4–P6	2.769266
P4–P8	2.682439

**Table 6:** Monitoring frequency in a day for the configurations P0–P2, P3–P6 and P2–P8.

Section	Monitoring Frequency in a Day
P0–P2	2.250252
P3–P6	1.875104
P2–P8	2.604552

**Table 7:** Summary table of monitoring frequencies for the different UAV responsibility zone allocation options. The values presented indicate the expected monitoring frequency in a day per drone. Highlighted cells indicate the monitoring frequency between neighbouring take-off and landing points, i.e., the option of using 8 drones in total. The table allows for determining the highest frequency of monitoring of the whole object.

		Landing								
		0	1	2	3	4	5	6	7	8
**Take-off**	**0**	–	4.70	2.25	1.51	1.14	1.00	0.80	1.33	1.22
	**1**	4.70	–	4.27	1.48	1.13	0.99	0.79	1.29	1.18
	**2**	2.25	4.27	–	4.62	2.34	1.81	1,25	3.12	2.60
	**3**	1.51	1.48	4.62	–	4.73	2.97	1,89	9.60	5.39
	**4**	1.14	1.13	2.34	4.73	–	7.98	2.77	3.17	2.68
	**5**	1.00	0.99	1.81	2.97	7.98	–	4.00	2.27	1.97
	**6**	0.80	0.79	1.25	1.89	2.77	4.00	–	1.58	1.43
	**7**	1.33	1.29	3.12	9.60	3.17	2.27	1.58	–	14.84
	**8**	1.22	1.18	2.60	5.39	2.68	1.97	1.43	14.84	–

**Table 8:** Expected number of maintenance cycles for each module of UAV used.

Component	Drone 0	Drone 1	Drone 2
Propellers	176.56	184.36	187.01
Batteries	19.25	18.13	18.85
Motors	9.17	9.14	9.22

## Data Availability

The data presented in this study are available on request from the corresponding author.

## Abbreviations

The following abbreviations are used in this manuscript:

UAV	Unmanned aerial vehicle
IEEE	Institute of Electrical and Electronics Engineers
FAA	Federal Aviation Administration
VTOL	vertical take-off and landing
IRMA	Interactive Reconfigurable Matrix of Alternatives
MCDM	Multiple-Criteria Decision Making
